# Household Diversity and the Impacts of COVID-19 on Families in Portugal

**DOI:** 10.3389/fsoc.2021.736714

**Published:** 2021-10-22

**Authors:** Rita Gouveia, Vasco Ramos, Karin Wall

**Affiliations:** Instituto de Ciências Sociais, Universidade de Lisboa (ICS-ULisboa), Lisboa, Portugal

**Keywords:** COVID-19 pandemic, family relationships, household diversity, linked-lives, social impacts, vulnerability

## Abstract

Throughout the world, the COVID-19 pandemic disrupted family routines, relationships, projects and sociability, threatening the health, income, social cohesion, and well-being of individuals and their families. Lockdown restrictions imposed during the first wave of the pandemic challenged the theories, concepts, and methods used by family sociologists and the intersecting fields of gender and social inequality. By restricting physical interactions to co-resident family members, the household regained a privileged role as a crucial social laboratory for studying the impact of COVID-19 on family life. The difficulties encountered by individuals in maintaining and dealing with close relationships across households and geographical borders, in a context in which relational proximity was discouraged by the public authorities, exposed the linked nature of family and personal relationships beyond the limits of co-residence. The main aim of this article is to investigate the social impacts of the pandemic on different types of households during the first lockdown at an early stage of the pandemic in Portugal. Drawing on an online survey applied to a non-probabilistic sample of 11,508 households between 25 and 29 March 2020, the authors combined quantitative and qualitative methods, including bi-variate inferential statistics, cluster analysis and in-depth case studies. The article distinguishes between different household types: solo, couple with and without children, extended, friendship, lone-parent families, and intermittent arrangements, such as shared custody. A cross-tabulation of the quantitative data with open-ended responses was carried out to provide a refined analysis of the household reconfigurations brought about during lockdown. The analysis showed how pre-existing unequal structural living conditions shaped the pathways leading to household reconfiguration as families sought to cope with restrictions on mobility, social distancing norms, and other lockdown measures. The findings stress that, in dealing with a crisis, multilevel welfare interventions need to be considered if governments are to cater to the differentiated social needs and vulnerabilities faced by individuals and families.

## Introduction

The COVID-19 pandemic disrupted family routines, relationships, projects and sociability in countries throughout the world, threatening health, income, social cohesion, and well-being. Individuals and their families were forced to find ways of dealing with feelings of uncertainty, insecurity and anxiety while engaging in collective displays of solidarity and altruistic actions.

When the pandemic hit Portugal, the government declared a state of emergency on 18 March 2020 and, as in other EU member states, imposed rules about staying at home, restricting mobility and the amount of time spent out-of-doors. During the first wave of the pandemic, schools and universities were closed, and distance-learning systems were implemented. Unlike other European countries, the Portuguese regulations did not propose “support bubbles” for those living alone or in lone-parent households (Long et al., 2020; Trotter, 2021). Physical interactions were limited to co-resident family members, and lockdown restrictions forced individuals to confine their sociability to relationships within the domestic space. Leaving home was allowed only for outings to buy essential goods and services, accessing public services and healthcare facilities, travelling to and from work, assisting dependent people, walking pets and engaging in solo physical activity. Telework became mandatory for economic sectors unable to operate otherwise, further restricting social contacts for a considerable population segment. A substantial part of the workforce was unable to work from home: “essential workers” included those in healthcare, transport, manufacturing and construction, as well as security personnel.

To frame how the COVID-19 pandemic affected Portuguese households, it is necessary to consider preceding circumstances and to highlight pre-existing vulnerabilities of families and the economy. The Portuguese economy was still recovering from the 2008–2014 financial crisis, mainly driven by the hospitality and tourism industries. Several indicators showed a positive trend, namely decreasing monetary poverty, lower levels of material deprivation and less economic inequality, contributing to an improvement in the well-being of families (Correia, 2020). Yet, while the economy was growing and unemployment was falling, the recovery process remained fragile: a significant number of jobs were precarious, of low quality or in the informal economy. For example, in 2019, 17.2% of the population was at risk of poverty after social transfers, amounting to 21.6% when accounting for those who suffered social exclusion (Eurostat, 2019). In-work poverty risk reached 10.9% among couples with dependent children and 26.4% for lone-parents. A recent study (Diogo et al., 2021), drawing on consolidated data from the EU-Survey of Income and Living Conditions, confirmed that women, younger workers, those with education levels below upper secondary education and families with children, especially lone-parents and couples with more than two children, were most at risk of poverty.

Research also showed how, after the financial crisis, the spread and intensification of job precarity impacted the economic survival, future outlooks and expectations of families, especially among younger generations (Carmo and Matias, 2019). As achieving residential autonomy became difficult, leaving the parental home was often postponed. According to Eurostat (2021), the Portuguese leave their parental home on average at 30 years old, ranking among the oldest top five countries, in sharp contrast to Scandinavian and Central European countries, in which the transition is completed, on average, before the age of 24. Furthermore, for many families, the cost of, and access to, housing had become a problem in the years leading to the pandemic, namely in metropolitan Lisbon and Porto and, especially, in more touristic areas, which were under mounting speculative pressures (Allegra and Tulumello, 2019; Cardoso, 2019).

This portrait of the uneven social landscape of Portuguese society before the pandemic “earthquake” provides an insight into how deep-rooted structural inequalities would be amplified, and how new vulnerabilities would emerge as a result of the economic, social and COVID-19 health crises.

The public health containment measures impacted family relationships. Lockdown and social distancing rules resulted in the household assuming (or rediscovering) its central importance in social life. The household became a social laboratory for studying the impact of COVID-19 on family life. This renewed interest in households is thought-provoking and perplexing for family sociologists and scholars from intersecting fields of gender and social inequality (Gouveia et al., 2021). On the one hand, family sociology has been moving away from the study of families based solely on co-residence criteria and from using the household as the only setting for empirical research on family practices (Morgan and Morgan, 1996; Wall and Gouveia, 2014; Widmer, 2010). On the other hand, the difficulties encountered by individuals in engaging in and maintaining close relationships beyond their household borders during lockdowns, in a context in which relational proximity was constrained, suddenly exposed the linked nature of family and personal relationships beyond household limits (Gouveia et al., 2021). As support bubbles were not applied in Portugal, “spread-out” families struggled to maintain contact with family members and other significant relations who did not share the same roof. Stay-at-home policies restricted physical contact to household members, penalising families living outside the normative model of co-residence, blood, and marriage, even accounting for allowable exceptions. The restrictions “left people painfully aware of how much their wellbeing is linked to others and how much they take for granted the ability to be with others” (Settersten et al., 2020, p. 5). This interdependent nature of close relationships is in line with Glen Elder’s (1994) linked-lives principle of life-course theories, which refers to “the interaction between the individual’s social worlds over the life-span family, friends, and co-workers. To a considerable extent, macrohistorical change is experienced by individuals through such worlds.” (Elder, 1994, p. 6) Thus, changes occurring in individuals’ life trajectories affect the lives of their meaningful others, and vice-versa.

In this article, social vulnerability in the pandemic context refers to individuals’ pre-existing social living conditions, which were already highly unequal in Portuguese society, their household arrangements during lockdown and the material and subjective impacts of the pandemic. Using the concept of configurations in family relationships (Widmer, 2010), reconfigurations of social vulnerability were identified under pandemic circumstances caused by the disruptions to families’ social relationships. The linked-lives framework of vulnerability was also employed to capture a multidimensional and relational process, rather than to focus on single outcomes (Spini et al., 2017). Assuming the interplay between social structure and human agency, a multilevel lens was adopted in this study to evaluate the role of different micro and macro factors on the intensity of social impacts on families, including the intersecting domains of employment, finance and education. Finally, a relational approach was adopted to examine family interdependencies within and across households, focusing on the coping strategies adopted through the reconfiguration of living arrangements when their lives were disrupted. These concepts are useful to understand how the pandemic destabilised individuals’ social embeddedness in their “normal” family and personal configurations.

In sum, this article investigates the social impacts of the COVID-19 pandemic on different types of households – solo, couple with and without children, extended, friendship, lone-parent, and intermittent, including shared custody arrangements – during the early stages of the pandemic in Portugal and the first lockdown. The authors show how individuals coped with the disruptions produced by the pandemic through the adoption of different types of lockdown strategies and pathways. The findings highlight the structural embeddedness of these strategies, accounting for the shaping role of gender, age, education, housing conditions, work, and family status.

## Methods

At the outbreak of the pandemic, scant information was available about the social impacts of the pandemic on the Portuguese population. Little was known about how individuals were coping with health concerns and threats, and how they were adjusting to mobility restrictions, social distancing norms and the imposition of lockdown measures. An online survey was set up by a multidisciplinary team of researchers from the Institute of Social Sciences (ICS-UL) and the University Institute of Lisbon (ISCTE-IUL) (Magalhães et al., 2020). The survey was launched immediately after the first lockdown had been declared in Portugal on 18 March 2020. Data collection took place between the 25 and 29 March 2020. The survey aimed to monitor the initial impact of the COVID-19 pandemic via a range of variables beyond immediate health and epidemiological concerns, including self-reported indicators of both material (financial consequences), and subjective impacts (difficulties in dealing with restrictions).

The survey was also a tool to address factors that shaped vulnerabilities and exacerbated social inequalities during the unfolding crisis. Survey design and sampling procedures were determined by the urgency of the pandemic, which meant that they were more hastily prepared than is usual in sociological research. The survey was publicised through the Institute’s online platforms: websites, Twitter and Facebook accounts and email distribution lists. Additional dissemination was achieved through mainstream media outlets and the researchers’ social media accounts.

The final sample consisted of 11,508 residents in Portugal. Due to the recruitment methods, the sample was non-probabilistic, limiting statistical inference and an accurate representation of all segments of society. Bias concerned geographical, educational and social class distribution, but not age or gender. Residents in the Greater Lisbon Area and large urban coastal areas were over-represented, as were those with a university degree. The sample contained large contingents of professionals and office workers and fewer routine manual and frontline service workers. Under-representation of the latter occupational groups, combined with the relatively small number of respondents with lower educational levels and living in rural inland areas, limited the research team’s ability to use the survey findings to address the worsening of poverty and social inequality among more invisible and vulnerable segments of society.

Given its relatively large size, the sample nonetheless allowed for a cross-cutting analysis of issues affecting households; it enabled associations to be made between variables that shaped social relations. Closed and open-ended questions were included, simulating a quasi-mixed-methods approach and adding depth to the understanding of the effects of the pandemic on individuals and families. Respondents were questioned about the impact of the pandemic on income, perceptions of resilience, difficulties in coping with government restrictions, family–work reconciliation, and professional activities.

The first phase of data analysis examined household diversity and the social impacts of the pandemic, using bi-variate inferential statistics and cluster analysis. Drawing on the open-ended questions, a close-up view of the different household pathways was obtained in the second phase by selecting cases exemplifying the strategies identified in the quantitative data. These included changes in household composition, such as adult children returning to the parental home, individuals in non-cohabiting relationships before the pandemic who started living with their partners, nuclear families sheltering an older relative to facilitate care.

## Results

The analytical strategy followed three steps: mapping and characterising the diversity of household composition during lockdown; categorising the main types of social impact on different types of households; identifying the main household lockdown strategies distinguishing between those who remained and those who moved to a different household.

### Household Diversity During Lockdown

Several of the open-ended questions asked about household composition, such as: “Who are you living with during this period of familial lockdown? Please specify if partner, daughter, in-laws, or other”. Cases were excluded from the analysis if the information provided was insufficient, inconsistent or unclassifiable, resulting in a reduction of the sample to 11,061 valid cases. Households were classified according to four criteria that are widely used by Portuguese sociologists: the number of individuals in each household; the type of bond between them (kinship, alliance/marriage, affinity); number and type of family units within each household; and the presence of non-family members (Wall, 2005; Wall et al., 2014).

For instance, single-family households were classified as “couples with children”, whether respondents mentioned living with parents and siblings or with a partner and children. Complex family household arrangements included multiple family households where several families live together or extended family households where a couple lives with kin beyond their offspring (parents, uncles, cousins) or with other non-kin (friends). Given the constraints on mobility imposed during the lockdown, the study was especially interested in individuals who moved between households, for example, post-divorce children whose parents shared custody. These cases were dubbed “intermittent” regardless of the types of households between which they moved.

**FIGURE 1:**
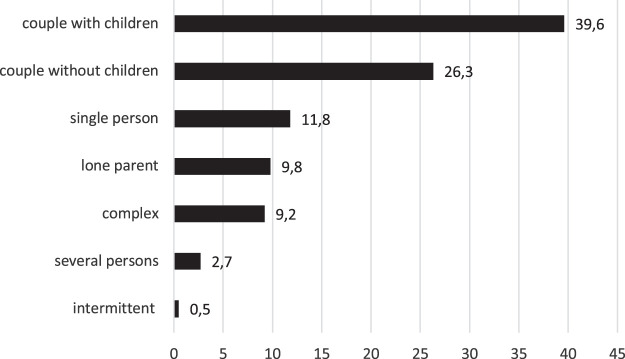
Household types during lockdown (%). Source: Impacto Social da Pandemia. Estudo ICS/ISCTE Covid-19. Note: *N* = 11,061.

The number of individuals per household, including the respondent, ranged from 1 to 12 people, with an average size of 2.80 and a standard deviation of 1.24. Figure 1 shows the distribution by type of household during the lockdown in the total sample.

Family households composed of couples with children were the most common type of household, with 40% of the cases. Childless couples accounted for a little over a quarter of the sample, while single-person households and lone-parent and complex families each accounted for about 10%. Only small percentages of respondents were living in households with non-related individuals or were in an intermittent situation.

Comparing this distribution with population data from the 2011 census shows a similar pattern (Wall et al., 2014). In 2011, 59% of the Portuguese population lived in a conjugal arrangement, split between couples with children (35%) and childless couples (24%). The percentages of lone-parent households, complex family households and several unrelated person households followed a very similar pattern to that in 2020, although single-person homes were slightly under-represented in the 2020 sample: 20% in the population in 2011.

### Social Impacts of the Pandemic on Households

This section begins with the characterisation of the socio-demographic profiles of individuals living in different households during lockdown using the following variables: sex, age, level of education, marital status, and current occupation (see Table 1). The analysis highlights salient features for each household type rather than exhaustively analysing the socio-demographic characteristics for each type.

**TABLE 1:** Sociodemographic features of household types during lockdown (in %).

		Single person	Lone-parent	Couple without children	Couple with children	Complex families	Several persons	Intermittent	Total
Gender	Male	36.4	27.7	**54.0**	42.6	38.3	34.3	46.7	42.8
	Female	**63.6**	**72.3**	46.0	57.4	**61.7**	**65.7**	53.3	57.2
	Total	100	100	100	100	100	100	100	100
Age group	16–24	1.9	**16.6**	2.1	**14.1**	**14.4**	**23.7**	3.3	10.0
	25–34	10.2	8.1	**16.6**	9.7	13.6	**31.0**	10.0	12.3
	35–44	17.1	17.0	15.2	**29.2**	19.3	16.3	**31.7**	21.7
	45–54	20.0	**33.3**	10.0	**32.3**	18.2	9.3	**30.0**	23.2
	55–64	**20.8**	**18.1**	**19.4**	11.5	**20.4**	9.3	**16.7**	16.1
	+65	**29.9**	7.0	**36.7**	3.3	14.2	10.3	8.3	16.8
	Total	100	100	100	100	100	100	100	100
Educational	Below Secondary Ed	1.6	2.0	**3.4**	1.9	**3.1**	1.3	3.3	2.4
	Secondary Ed	9.3	**17.1**	9.5	**15.5**	**17.4**	12.0	8.3	13.4
	University Degree	**89.1**	80.9	**87.1**	82.6	79.5	86.7	88.3	84.2
	Total	100	100	100	100	100	100	100	100
Marital Status	Single	**50.2**	**46.6**	12.5	23.0	30.2	**80.0**	30.0	28.0
	Married/Civil Part	5.1	8.6	**84.4**	**75.2**	57.6	5.7	36.7	59.1
	Divorced/Separated	**34.1**	**39.5**	2.6	1.7	10.7	10.7	**33.3**	10.7
	Widowed	**10.5**	**5.3**	0.4	0.1	1.6	**3.7**	0.0	2.1
	Total	100	100	100	100	100	100	100	100
Occupation	No longer working	**27.3**	10.1	**29.7**	6.5	14.6	15.0	8.3	16.4
	Working as before	**7.2**	5.6	5.2	**6.5**	6.2	4.0	8.3	6.1
	Tele-working	44.7	53.2	45.6	**56.9**	46.2	46.0	**60.0**	50.8
	Furlough/redundancy	1.8	1.3	1.2	1.5	**2.4**	**3.0**	1.7	1.6
	Student	2.7	**14.0**	2.2	**12.4**	**11.1**	**17.7**	3.3	8.7
	Other	16.3	15.8	16.2	16.2	**19.5**	14.3	18.3	16.4
	Total	100	100	100	100	100	100	100	100

Source: Impacto Social da Pandemia. Estudo ICS/ISCTE Covid-19.

Notes: Statistically significant associations are presented in bold, based on standardised residuals. The Qui2 values for the associations between all the socio-demographic variables and the type of household are statistically significant.

As illustrated in Table 1, single-person households were mostly composed of women, single, divorced and widowed persons, highly educated individuals (89.1% with a university degree) and people aged 55 or over. In most cases, respondents living in this household arrangement did not experience significant changes to their occupational status during the initial stage of the pandemic. Most of the respondents who were classified as being economically active continued working as before the lockdown. A significant proportion of respondents in this type of household were inactive because they had retired (27% compared to 16% in the total sample).

Lone-parent households included a high proportion of teenagers and young adults aged 16–24 of both sexes, and women aged 45–54 and 55–64. Within this household type, individuals with secondary-level education were over-represented, as were singles, divorcees and widowers. The high proportion of students (17% compared to 10% in the total sample) is associated with the over-representation of individuals aged 16–24 years.

Men were over-represented in households composed of couples without children, as were respondents aged 25–34 and over 55. In terms of educational attainment, individuals living in this type of household were often university graduates or had education below secondary level. Most were married or in a civil partnership. The majority of those of working age were working remotely.

Households composed of couples with children contained more men and women aged 35–54 and teenage/young adults who had generally completed secondary education and were married or living in a *de facto* partnership. They were over-represented among those working remotely or involved in distance learning.

Households comprising several unrelated persons tended to be younger. Women and single persons were equally over-represented. Individuals living within this household type were disproportionately affected by job loss or were on “gardening leave”, the precursor of furlough schemes in Portugal, the use of which grew exponentially as the pandemic hit Portugal.

Women were over-represented in complex family households, as were younger and older respondents aged 15–24 and 55–64, and individuals with educational levels below a university degree. This household type contained a significant proportion of individuals who were forced to take time off work. A large proportion of divorced/separated individuals in intermittent households were also found in this type of household.

To understand the diversity of social impacts in different segments of society, social profiles were mapped according to their material and subjective effects by carrying out a cluster analysis. Material impacts encompassed unintended changes in working and living arrangements. The subjective aspect focused on individual perceptions of lockdown rules, specifically concerning difficulties in dealing with mobility restrictions and self-assessment of personal resilience in coping under these conditions. Given the social structure of Portuguese society, our sole hypothesis was that the pandemic crisis would heighten pre-existing social inequalities along the lines of gender, age, social class, employment, and family conditions, translating into different social profiles of impact. A two-step cluster method was applied, which is a well-suited and robust technique to use with categorical variables (Chiu et al., 2001). The seven variables analysed are listed in Table 2.

**TABLE 2:** Variables included in clustering of social impacts of the pandemic.

Variable	Categories
Level of difficulty in dealing with the restrictions	Easy
	Difficult
Assessment of current income level	Comfortable
	Acceptable
	Insufficient (struggling)
Financial impact of the pandemic	Financial situation affected
	Financial situation not yet affected
Occupation/work during the pandemic	Not working before
	Working under the same conditions
	Teleworking
	Studying
	Dismissed/forced vacation
	Others (including furlough)
Housing conditions	Satisfactory/adequate
	Reasonable/inadequate
Expectations concerning the extent of lockdown	Short-term
	Medium-term
	Long-term
	Don’t know
Personal resilience for coping with restrictions	Low
	Average
	High
	Don’t know

The cluster analysis enabled the research team to identify the main characteristics of vulnerable groups, regardless of population weight, which helped to compensate for the sampling bias. Four impact profiles were identified through a four-cluster solution, combining statistical robustness and sociological interpretability.

Table 3 shows that the dominant profile consisted of Materially Vulnerable–Subjectively Constrained individuals. Most respondents in this group mentioned finding it difficult to deal with lockdown restrictions. They expected them to be short term and often alluded to their own low resilience. Significantly, most respondents within this group, particularly furloughed workers, and distance learning students, were already suffering from difficult material conditions, either because their incomes had fallen, or their housing conditions had deteriorated. This profile is thought to apply to a much higher proportion of the Portuguese population.

**TABLE 3:** Four profiles of social impact (based on distribution of active variables) (in %).

		Total (*N* = 11,333)	Materially vulnerable–subjectively constrained (*N* = 3,322)	Materially comfortable–subjectively relaxed (*N* = 3,133)	Materially comfortable–subjectively relaxed (*N* = 2,754)	Materially fragile–subjectively uncertain (*N* = 2,124)
	Size	100.0	29.3	27.6	24.3	18.7
Level of difficulty in dealing with restrictions	Easy	68.3	48.0	**100.0**	58.3	66.6
	Difficult	31.7	**52.0**	0.0	**41.7**	**33.4**
	Total	100	100	100	100	100
Assessment of current income level	Comfortable	45.2	15.9	**58.9**	**100**	0.0
	Acceptable	44.6	**58.9**	41.1	0.0	**85.4**
	Insufficient (struggling)	10.1	**25.2**	00.0	0.0	**14.6**
	Total	100	100	100	100	100
Financial impact of the pandemic	Financial situation affected	23.6	**59.4**	00.5	8.2	21.7
	Financial situation not yet affected	76.4	40.6	99.5	91.8	78.3
	Total	100	100	100	100	100
Work during the pandemic	Not working before	16.3	09.5	18.5	17.9	**21.5**
	Working under the same conditions	06.1	06.6	05.1	05.4	**07.9**
	Teleworking	51.1	45.7	**55.8**	**54.8**	47.6
	Studying	01.6	**03.7**	00.7	00.4	01.0
	Dismissed/“gardening leave”/furlough	08.7	**10.7**	**10.2**	07.7	04.8
	Others	16.2	**23.8**	09.6	13.8	17.3
	Total	100	100	100	100	100
Housing conditions	Satisfactory/adequate	85.8	70.2	**97.4**	**95.6**	80.5
	Reasonable/inadequate	14.2	**29.8**	02.6	04.4	**19.5**
	Total	100	100	100	100	100
Expectations concerning the extent of lockdown	Short-term	27.8	**39.9**	**47.7**	11.7	00.0
	Medium-term	33.6	**47.0**	**52.3**	21.9	00.0
	Long-term	20.5	07.6	00.0	**37.5**	48.7
	Don’t know	18.2	05.4	00.0	28.9	**51.3**
	Total	100	100	100	100	100
Personal resilience for coping with restrictions	Low	43.8	**57.7**	**61.4**	28.6	15.6
	Average	16.1	13.6	**22.8**	15.0	11.6
	High	06.7	02.7	01.4	**12.3**	**13.5**
	Don’t know	33.4	26.0	14.4	**44.1**	**59.3**
	Total	100	100	100	100	100

Source: Impacto Social da Pandemia. Estudo ICS/ISCTE Covid-19.

Note: Statistically significant associations are presented in bold (based on standardised residuals).

Materially Comfortable-Subjectively Relaxed individuals made up the second-largest profile. These respondents stated they had no difficulty dealing with lockdown restrictions, which they expected to be short to medium term, and were confident in their resilience to cope with the situation. Unlike respondents in the first profile, most experienced no change in their standard of living and were in better housing conditions than the first group. They were mostly teleworkers, long-distance students and pensioners.

A third profile was composed of Materially Comfortable-Subjectively Constrained respondents. They expected restrictions to last longer and found them harder to endure. They had not experienced any significant material impacts on their incomes and were living in good housing conditions. They were also mainly teleworkers, long-distance students and retired persons.

The fourth profile comprised the Materially Fragile-Subjectively Uncertain respondents. They expressed uncertainty both in terms of duration and personal resilience in the face of lockdown restrictions. They also reported having difficulty dealing with the situation, albeit less so than those with a Materially Vulnerable-Subjectively Constrained profile. Most respondents in this group mentioned that, while they were not suffering financially, their material conditions were, at best, reasonable. They had been able to maintain their previous occupational status: they had already been out of work or continued working in the same conditions.

Table 4 shows the associations between household composition during lockdown and the social profiles of material and subjective impacts.

**TABLE 4:** Profiles of social impact by household type (in %).

	Single person	Lone parent	Couple without children	Couple with children	Several persons	Complex	Intermittent	Total
Materially comfortable-subjectively constrained	**26.3**	20.2	**26.6**	24.4	22.8	19.7	**33.3**	24.4
Materially vulnerable-subjectively constrained	23.1	**33.9**	24.7	**31.2**	**40.9**	**33.8**	30.0	29.3
Materially comfortable-subjectively relaxed	28.3	25.0	**30.8**	27.2	21.5	26.1	20.0	27.8
Materially fragile-subjectively uncertain	**22.3**	**20.9**	17.9	17.2	14.8	**20.4**	16.7	18.6
Total	100	100	100	100	100	100	100	100

Source: Impacto Social da Pandemia. Estudo ICS/ISCTE Covid-19.

Note: Statistically significant associations are presented in bold (based on standardised residuals).

Respondents living in complex families and several-person households were over-represented among the materially and subjectively most vulnerable group. This finding also holds for respondents in lone-parent and couple households with children. Conversely, a high proportion of childless couples were found among respondents who displayed comfortable material and subjective living conditions. Respondents in the materially comfortable but highly constrained group in terms of their subjective experiences accounted for a higher proportion than expected of those living alone, in couples without children and transiting between different households. Complex families, lone-parent and single-person households were over-represented among respondents who were gripped by feelings of uncertainty when dealing with minor material constraints.

Table 5 shows the number of household members and the number of minors living at home during lockdown. Those who were materially and subjectively vulnerable were over-represented in households with more than four members, as well as in families co-residing with children and teenagers. Again, this seems to reinforce the previous observation that families in complex multigenerational households, as well as those families living with under-aged children, such as lone-parent and couple with children households, stand out as materially and subjectively more vulnerable. Household members from different generations were affected by the pandemic when they were going through specific life-course phases, which then affected the whole family. This observation shows the linked lives aspects of vulnerability, and how crucial it is to situate the pandemic’s impacts within the life courses of individuals. The two profiles, which represent those who were in more comfortable positions regarding their financial and material living conditions, were strongly associated with smaller households (fewer than two persons) and without young children.

**TABLE 5:** Profiles of social impact by household size and number of under-aged household members during lockdown (in %).

	Materially comfortable-subjectively constrained	Materially vulnerable-subjectively constrained	Materially comfortable-subjectively relaxed	Materially fragile-subjectively uncertain	Total
Household size (number of residents including the respondent)					
1 person	**13.1**	9.6	12.3	**14.7**	12.1
2 persons	**36.9**	31.5	**36.9**	34.4	34.9
3 persons	21.9	26.9	22.4	24.2	23.9
4 persons	20.3	**22.0**	21.2	17.9	20.6
5 + persons	7.8	**9.9**	7.2	8.8	8.5
Total	100	100	100	100	100
Number of under-aged household members					
0 person	**64.3**	57.0	**65.8**	62.8	62.3
1 person	16.2	**20.5**	15.3	16.6	17.3
2 persons	12.5	**14.1**	12.5	12.2	12.9
3 + persons	7.0	**8.4**	6.4	**8.3**	7.5
Total	100	100	100	**100**	100

Source: Impacto Social da Pandemia. Estudo ICS/ISCTE Covid-19.

Notes: Statistically significant associations are presented in bold, based on standardised residuals.

In sum, the most vulnerable respondents were those living in complex families and lone-parent households during lockdown, followed by those who live in couples with children, particularly young children, and those cohabiting with several unrelated persons. Couples or those living alone were strongly associated with more comfortable material circumstances, but subjectively their attitudes were more mixed, encompassing both those who had a more relaxed attitude and those who were apprehensive or even felt constrained. Overall, vulnerability seems to be associated with the number of household members and the presence/absence of young people.

### Changing Household Composition: Before and After Lockdown

While most respondents remained in their usual household during lockdown, roughly 10% (1,150) experienced a change in household composition. Different types of reconfigurations represent distinct coping strategies for dealing with lockdown restrictions. This section examines the pathways between households followed by individuals living in different household structures.

The comparison of average household size before and after lockdown did not reveal a statistically significant difference: before lockdown Mean = 2.87; during lockdown Mean = 2.83. This apparent stability is misleading since household size says little about changes in household composition. When the breakdown of household types is compared before and during lockdown (Table 6), the proportion of complex households increases, hinting that family structures tended to diversify. This growth is complemented by a decrease in the proportion of single-person households and couples without children. By contrast, the size of some household types shrank as the number of lone-parent families increased and households composed of several unrelated individuals decreased.

**TABLE 6:** Distribution of household types after and during lockdown (in numbers and %).

Before lockdown			During lockdown		
	Frequency	Percent		Frequency	Percent
Single person	1,438	13.0	Single person	1,306	11.8
Lone parent	1,027	9.3	Lone parent	1,084	9.8
Couple without children	3,032	27.5	Couple without children	2,913	26.3
Couple with children	4,284	38.8	Couple with children	4,386	39.6
Several persons	433	3.9	Several persons	300	2.7
Complex	733	6.6	Complex	1,013	9.2
Intermittence	88	0.8	Intermittence	60	0.5
Total	11,035	100	Total	11,062	100

Note: Statistically significant associations are presented in bold (based on standardised residuals).

Source: Impacto Social da Pandemia. Estudo ICS/ISCTE Covid-19.

However, merely comparing the two points in time says little about the direction of change since it applies to all household types. This section focuses on the subsample of respondents who experienced a shift in their household structure (*N* = 1,150) and their different pathways. A cross-tabulation of these data with open-ended responses was required to enable a more refined analysis of the data.

For respondents living alone before lockdown who experienced a change in their household structure, three main pathways were observed in the responses to the open-ended questions in the survey. As illustrated in Table 7, close to a third of over 18-year-olds moved in with a parent or took them into their home. Such decisions were often taken to facilitate caring for older people, sick or partially dependent parents. According to one respondent: “I have sheltered my mother at my place since she is sick, and I feel that she has a lot of limitations in getting access to healthcare.” (F, aged 37).

**TABLE 7:** Distribution of household types that changed during lockdown (in %).

	Household composition before lockdown								
		Single person	Lone-parent	Couple without children	Couple with children	Complex families	Several persons	Intermittent	Total
Household composition during lockdown	Single person	0.0	19.3	9.3	6.4	6.3	11.3	29.4	8.3
	Lone-parent	**30.3**	**21.6**	03.8	5.6	04.7	09.2	**23.5**	13.0
	Couple without children	**19.7**	09.1	01.7	7.7	**21.9**	08.7	11.8	10.2
	Couple with children	26.9	13.6	**46.0**	22.2	15.6	**46.2**	05.9	31.1
	Complex families	12.0	30.7	**38.0**	**54.3**	**48.4**	15.4	23.5	31.4
	Several persons	10.7	05.7	01.3	3.8	03.1	**09.2**	00.0	05.7
	Intermittent	0.40	00.0	00.0	00.0	00.0	00.0	**05.9**	00.3
	Total	100	100	100	100	100	100	**100**	100

Note: Statistically significant over-representations are presented in bold (based on standardised residuals).

Another route involved individuals in non-cohabiting relationships before the pandemic who started living with their partner (19.7%). In this case, the pandemic triggered cohabitation for partners who had not lived together before. Another respondent commented: “I felt the urge to live together with my partner. If I had remained alone, it would have been more difficult to cope with lockdown.” (F, aged 74) Finally, a third major scenario was the formation of either a new complex or extended family household shared with an assortment of family members of different generations (grandparents, cousins, uncles) (12%), or co-residence of several unrelated family members (10.7%). In the first case, care issues often motivated the change:

I decided to remain isolated at my grandmother’s house to guarantee that I could provide her support. Also, I wanted to manage her physical contacts with others and to adapt her house to this critical phase*.* (F, aged 56)

Respondents who changed from living in lone-parent households before lockdown followed three main strategies. In some cases (22%), they remained in the same type of household, but with a smaller number of children, as explained by one respondent: “I’m living with my son, but I used to live with two more daughters.” (F, 44 aged) In other cases (19.3%), respondents decided to live apart from their children, or more rarely from their parents in the case of young adults. These changes were made to protect themselves and others from COVID-19 infection and were often made by those who were professionally involved in healthcare, public security forces and related services:

I work in the security forces, and I continue to work [away from home]. I’m alone, since my job is considered a high-risk activity, and my son is staying at his father’s house for safety reasons. (F, aged 46)

Another possibility was to expand the household to include other kin members (12%). Circumstances again expedited the change due to concerns about care arrangements: “My mother became bedridden with Alzheimer’s, and I need four persons daily to help me take care of her.” (F, aged 70)

For those who were living as a couple without children before lockdown, two main solutions were adopted. A large proportion (46%) reverted to a nuclear family structure, either welcoming children back into their homes or moving in with them:

The management of time has changed since I have sheltered my two adult sons at home. They were living abroad, and they have now returned due to the pandemic. The house where we live is small. (F, aged 52)

In other cases (38%), the household grew to accommodate an extended family, as other relatives moved in:

I’m retired, but my wife and children are still working remotely. My granddaughter is attending classes from home. We provide support to my 90-year-old mother and my 89-year-old father-in-law. (M, aged 66)

Two main pathways were followed by respondents who lived in extended families before the pandemic. In many cases, the household expanded as more family members were added to the fold (48.4%), often leading to even more complex family configurations. These arrangements did not necessarily have negative connotations, although they could lead to ambivalent experiences:

Since I am with my family in a big space, with a child, it almost feels like being on holiday. However, what hurts me most is knowing that my father is institutionalised. (F, aged 64)

Another route was “household de-complexification”, where individuals began to live as couples without children (21.9%). Such was the case of three-generation households, in which grandparents lived with their grandchildren or with adult children before, but where social distancing measures imposed separation:

The hardest thing is to be away from my little grandchildren. We have been very present grandparents, and now we are prevented from providing them with support. (F, aged 70)

For those who were living as a couple with children**,** forming a new extended household was the most common pathway (54.3%). In some cases, this option facilitated a trade-off in care:

The main difficulty is to reconcile work and the attention I need to give to my two daughters. The fact that my mother came to my house mitigated that problem. (M, aged 39)

In other cases (22.2%), even though the family’s structure remained the same, a son or daughter returned home, which constituted a “non-scheduled” life transition precipitated by the pandemic. This is an example of a reconfiguration of both individuals’ life trajectories and their networks of meaningful relationships within and beyond household borders.

Among those who lived with several unrelated persons before the pandemic, moving back to a nuclear family was the most common pathway during lockdown (46.2%). Young people who left an apartment shared with friends to return to their parental home often experienced a difficult homecoming. One respondent commented: “Dealing with my family every day is boring!” (man, aged 19) Smaller but statistically significant numbers of respondents started living alone (11.3%) or remained in the same type of household (9.2%) but with fewer people:

I usually live in a students’ residence. The only thing I miss is the social gatherings/conviviality, since all my flat mates have returned home, and I was the only one who stayed. (M, aged 22)

Among those in intermittent households before the pandemic, mainly parents with shared custody arrangements, the most common pathway was to start single living (29.4%) with its incumbent problems, meaning, according to one respondent: “being away from my daughter who is with the father … and 100 km apart, [plus] taking meals to my parents every day, who live in two different homes … ” (F, aged 42). This pathway was most frequently adopted by lone parents (23.5%), whereby some children remained with one of the parents, usually the one who represented less risk and whose housing and care conditions were more suitable. Another situation that triggered this route was adopted by those who relied on day-care facilities that ceased to operate:

The most difficult thing is to take care of my dependent adult son, without having any support from the institution where he was a beneficiary and being unable to go outside and visit my family. (F, aged 70)

In sum, at the early stage of the COVID-19 pandemic, respondents followed a plurality of household “reconfiguration pathways” as a coping strategy to enable them to face the disruption and difficulties created by the first lockdown and restrictions on mobility and physical contact. These household reconfigurations operated in two main directions: towards nuclearisation or greater complexity of living arrangements. In other situations, strategies involved self-isolation or intermittence between different households.

## Discussion and Conclusion

These findings provide a diverse landscape of household (re)configuration during the initial lockdown in Portugal after the onset of the pandemic. They show that changes in household living arrangements are associated with different social profiles and have differential material and subjective impacts. Households reconfigured to cope with restrictions on mobility, social distancing norms, and other lockdown measures in the context of pre-existing unequal structural living conditions. These responses challenged the linked-lives nature of the social relationships in which individuals were embedded. Although the survey was carried out during the early stage of the pandemic, results show that individuals were already feeling an impact on their financial, housing and working conditions. They also expressed concern about how they would handle restrictions.

Respondents in complex families, lone parents and couples with young children, as well as individuals living with non-related people during lockdown, faced a higher risk of experiencing deterioration in both their material and subjective living conditions. These household types were more strongly associated with groups already materially vulnerable and fragile before the pandemic. Respondents who lived alone or with one partner were over-represented in two contrasting profiles: those who felt apprehensive or constrained; those who reported more difficulty dealing with lockdown restrictions. Many of them were contemplating an uncertain long-term outcome with low resilience for enduring restrictions. Other respondents were coping well with the lockdown and restrictions, anticipating a more positive outcome and manifesting an easy-going attitude towards confinement, mobility and social distancing norms. The most vulnerable and fragile social profiles were over-represented among larger households and in households with young children and teenagers.

The survey findings highlighted the diversity of the coping strategies developed during the early stage of the pandemic through household reconfiguration and the disruption it caused to their normally linked lives. Family nuclearisation occurred when grandparents who previously looked after grandchildren on weekdays became isolated. Due to the loss of financial and material resources, some adult children were forced to return to the parental home. Reintegration into their families of origin was often experienced as a stressful transition. This trend is in line with the findings from a UK survey conducted during the first lockdown, which shows how this return to the parental nest was associated with high stress levels reported by both adult children and their parents (Evandrou et al., 2021). Some of the alternative solutions adopted led to the complexification of households, when for example, an adult child or a nuclear family sheltered elderly relatives to facilitate care, thus forming multigenerational households. These household changes led to a reconfiguration of social linkages in existing family living arrangements. Some respondents opted for self-isolation, for example, health professionals who decided to live alone to protect co-resident family members. Another coping strategy was intermittence, as individuals moved between households, which was the route adopted by some post-divorce families already before the pandemic, but which was consolidated during the lockdown.

Faced with an alarming unforeseen disruption to their lives, respondents followed strategies ultimately aimed at protecting their families and themselves, even if it meant sacrificing independence or separating from loved ones. While all solutions depended on realistic possibilities and priorities, they consistently demonstrated that lives are inexorably linked. Sociologically, this reminds us of the importance of diversity in family configurations. “Doing family” overrides the limits of the household, but co-residence is still a major factor in family relational proximity. A key finding for policy design is the need to broaden the notion of significant family members beyond the normative models of co-residence, for example by including support bubbles that emerged in the pandemic both as a new term and as a practice.

Studying change in household composition continues to be a productive means of understanding what happens within families. Our analysis shows that the decision of many individuals to reunite with parents or adult children confirms the ideological strength of the notion of family and its material and emotional role in sheltering individuals in times of economic hardship, and social and emotional upheaval. These reconfigurations of living arrangements also highlight the importance of intergenerational family relationships in care provision. Different types of support exchange between generations were identified, such as adult children taking care of their elderly parents, often in a “sandwich” position, as they also cared for their grandchildren. Due to school closures, some grandparents moved to take care of their grandchildren, supporting their adult children who had to contend with employment responsibilities, thereby forming extended living arrangements. The literature shows how intergenerational relationships have long been characterised by support, solidarity and ambivalence, tension and conflict (Brannen et al., 2004; Connidis, 2015; Girardin et al., 2018; Lüscher, 2002). The pandemic added another layer to the equation: the risk of transmitting the virus between generations discouraged face-to-face contact, on the one hand, and confirming the need to provide support to vulnerable older adults, on the other.

The study contributes to understanding processes of (re)configuration of social vulnerability in the context of an unprecedented health crisis. The heuristic concept of linked lives served as an analytical framework for studying social vulnerability and capturing the multidimensionality of the material and subjective impacts of the pandemic, as well as the differentiated needs and vulnerabilities in each type of living arrangement during the pandemic. As Elder et al. (1985, p. 40) stressed decades ago: “Each generation is bound to fateful decisions and events in the other’s life course.” Thus, the concept enabled us to understand the interdependent nature of the pandemic effects and to tackle both the socially differentiated contexts and the relational shaping processes of both fragility and resilience.

The triangulation of the empirical quantitative and qualitative material afforded a “zoom in” and “zoom out” perspective with the qualitative material confirming, complementing, elaborating or contradicting the trends and results generated by quantitative analysis (Brannen, 2005). The study also provided important insights regarding the problems of “doing research” during an ongoing pandemic. It showed how urgency in gathering information affects sampling procedures, questionnaire design and, ultimately, the external validity of findings.

Pre-existing social inequalities and the multidimensionality of the social impacts of the COVID-19 pandemic call for multilevel welfare interventions and policy designs to address housing conditions, family–work reconciliation, gender equality, employment and income protection, as well the need for more robust investment in policy regimes of care and support targeted at older and young people.

From an early stage in the pandemic, health containment and mitigation efforts were paired with policy measures to support families and companies and maintain employment levels. For families, these measures encompassed, among others, direct financial support for caretaker parents of children aged up to 12 years, subsidies to those who needed to self-isolate and automatic renewal of social benefits or legal status in the case of migrants (Correia, 2020). Additionally, school canteens were kept open, serving takeaway meals for children who qualified for free school meals, although eligibility rules were not altered to account for an increase in the number of children at risk of poverty.

With the aim of avoiding unemployment, the Portuguese government created a “simplified furlough” scheme, along with exceptional support measures for self-employed, small-company managers of companies forced to stop or significantly reduce operations, and later for independent workers in cultural and recreational industries. Companies were also temporarily exempted from paying social security, taxes and other capital-related expenses (Caldas et al., 2020). A moratorium on mortgages was declared to prevent defaults and enable suspension of payment for 6 months. Measures such as these were extended or fine-tuned to mitigate the effects of the second and third waves of the pandemic.

Initial assessments suggested that those in less protected and low paid jobs, more often women and younger workers, were likely to suffer the brunt of the economic impact of the pandemic in Portugal (Almeida and Santos, 2020). Overall, in terms of employment, the immediate effect was felt both in decreased activity rates and spikes in unemployment, especially during the second and third trimesters of 2020 (MTSSS, 2020). Some of these initial impacts started to subside by the fourth trimester of 2020 (MTSSS, 2021). While employment protection measures had some success, accessing the labour market became increasingly difficult. The number of families applying for Social Insertion Income (the last line of social benefits) also increased. Subsequent analysis confirmed that a segmented labour market, where employment protection varies depending on insider−outsider status, contributed heavily to a magnified, asymmetric and gendered shock (Nunes et al., 2021; Peralta et al., 2021). The gendered bias in the effects of the pandemic is related to paid work, as women constitute the majority of frontline workers in healthcare and social work (Correia, 2020). But women’s unpaid work was also affected since women were more often overloaded with domestic and care work for children, older and other dependents, a situation exacerbated by the closure of services during the initial stages of COVID-19.

Despite the early timing and biases inherent in the survey, the socially structured differences in impact that it revealed afford warnings for policymakers dealing with future impacts of the pandemic on vulnerable segments of society. The initial survey was a snapshot that captured the immediate reactions to the onset of the pandemic. A similar online survey carried out during the second lockdown in February 2021 pointed to the longer-term effects of the pandemic crisis (Gouveia et al., 2021). In the first lockdown, narratives revolved around fear, uncertainty and the unknown, often concerning employment and financial security. In the 2021 survey, an additional layer of fatigue and fragility to already difficult living conditions surfaced, often affecting mental health and calling for urgent policy interventions.

## Data Availability

The data are available with open access on request, and on the establishment of an agreement between the users and the survey coordinators to guarantee the ethical requirements of responsible scientific research.
